# Effects of racket moment of inertia on racket head speed, impact location and shuttlecock speed during the badminton smash

**DOI:** 10.1038/s41598-023-37108-x

**Published:** 2023-08-28

**Authors:** H. Towler, S. R. Mitchell, M. A. King

**Affiliations:** 1 School of Sport, Exercise and Health Sciences, Loughborough University, Loughborough, LE11 3TS UK; 2 Electrical and Manufacturing Engineering, Wolfson School of Mechanical, Loughborough University, Loughborough, UK

**Keywords:** Engineering, Applied physics

## Abstract

How the racket properties impact performance of the badminton smash is relatively unknown, and further insight could help players/coaches select the most appropriate racket. Three-dimensional position data of the racket and shuttlecock were collected (500 Hz) for 20 experienced badminton players performing a series of forehand smashes with five swingweight (${I}_{s}$) perturbed rackets, ranging from 85–106 kg·cm^2^. ${I}_{s}$ was calculated using a balance board and simple pendulum method, and modal analysis was performed using laser vibrometry to capture the fundamental frequency and distal node location for each racket. As ${I}_{s}$ increased a reduction in racket head speed was found with on average a 0.7 m·s^−1^ decrease per 5 kg·cm^2^ increase in ${I}_{s}$, however this did not lead to slower shuttlecock speeds. The impact location tended to move closer to the tip as the fundamental frequency node moved closer to the tip (as ${I}_{s}$ increased), providing some evidence that participants may subconsciously strike the shuttlecock at the node location to provide desirable sensory feedback. The increase in racket head speed but not shuttlecock speed was likely due to the distal increase in longitudinal impact location as ${I}_{s}$ increased, as well as an increase in effective mass for a given impact location. Additionally, removal of the deformation component (additional racket head speed due to the racket noticeably bending and recovering) of racket head speed increased the effect size of the relationship with ${I}_{s}$, where rackets with greater ${I}_{s}$ had larger deformation velocities. The research provides further insight into the smash performance characteristics of experienced badminton players, particularly based on racket properties. Further research is required to confirm the coincidence between node location and longitudinal impact location.

## Introduction

The badminton smash produces the fastest object velocity in any racket sport, with shuttlecock speeds as high as 107 m·s^−1^ reported in the literature^1^, achieved with racket head velocities as high as 71.5 m·s^−1^, measured normal to the stringbed plane at the racket head centre^2^. Understanding how the properties of the racket influence performance of the badminton smash is of interest to players and coaches.

**Figure 1:**
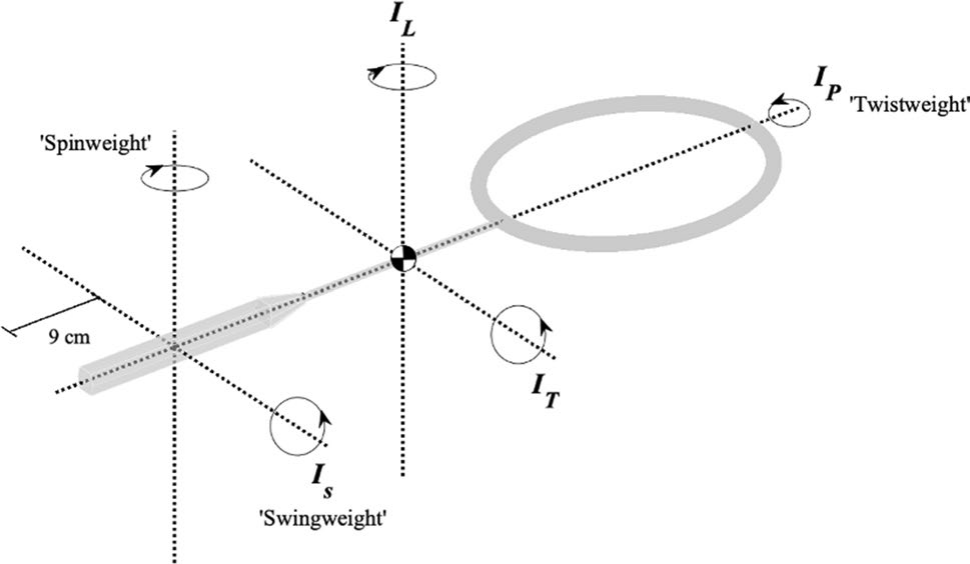
Mass moments of inertia of a badminton racket. Principal moments of inertia (${I}_{T}, { I}_{L}, {I}_{P}$) refer to the transverse, lateral and polar axes, and common terms for different moments of inertia are shown.

Moment of inertia (MoI) is an important racket parameter, in particular the MoI about an axis near the handle end where the racket is gripped and parallel to the stringbed, often termed the ‘swingweight’ axis. The location of this axis (the distance from the handle end) varies dependent upon the sport and implement. This distance is commonly 9 cm, 10 cm (4 inches) and 36 cm (14 inches) from the handle end for badminton rackets, tennis rackets and golf clubs, respectively. Hereinafter, the MoI about this axis will be termed ${I}_{s}$. Common MoI terms used within racket sports are shown in Fig. 1. It should be noted that the location of the ${I}_{s}$ axis is arbitrary and could differ between players and strokes. Typical ${I}_{s}$ values of badminton rackets are 90–97 kg cm^2^ and masses approximately 0.085–0.095 kg^2,3^.

Past research has shown an inverse relationship between ${I}_{s}$ and implement swing speed during the badminton smash^3^, tennis serve^4,5^ and golf drive^6^, however, these reductions in swing speed did not affect outbound object speed. This could be due to a combination of an increase in effective mass as ${I}_{s}$ increases (for a given impact location) and differences in impact location^3,5^. Previous research has found that in tennis the impact location coincides with the node location^7,8^, where the node location moves up the racket with more mass placed distally^9^. Schorah et al*.*^10^ concluded that an implement with greater ${I}_{s}$ typically results in a lower swing speed, and for a given swing speed, an implement of greater ${I}_{s}$ produces a higher post-impact object velocity.

This study aimed to investigate relationships between ${I}_{s}$ and racket head speed, impact location and post-impact shuttlecock speed within a cohort of experienced badminton players.

## Methods

A series of mechanical tests were performed to gain full understanding of the racket mechanical properties (Stage 1). Subsequently, performance testing with participants using motion capture was conducted to understand the effects of racket properties (moment of inertia) on various badminton smash performance parameters (Stage 2).

### Stage 1: Racket preparation

**Figure 2:**
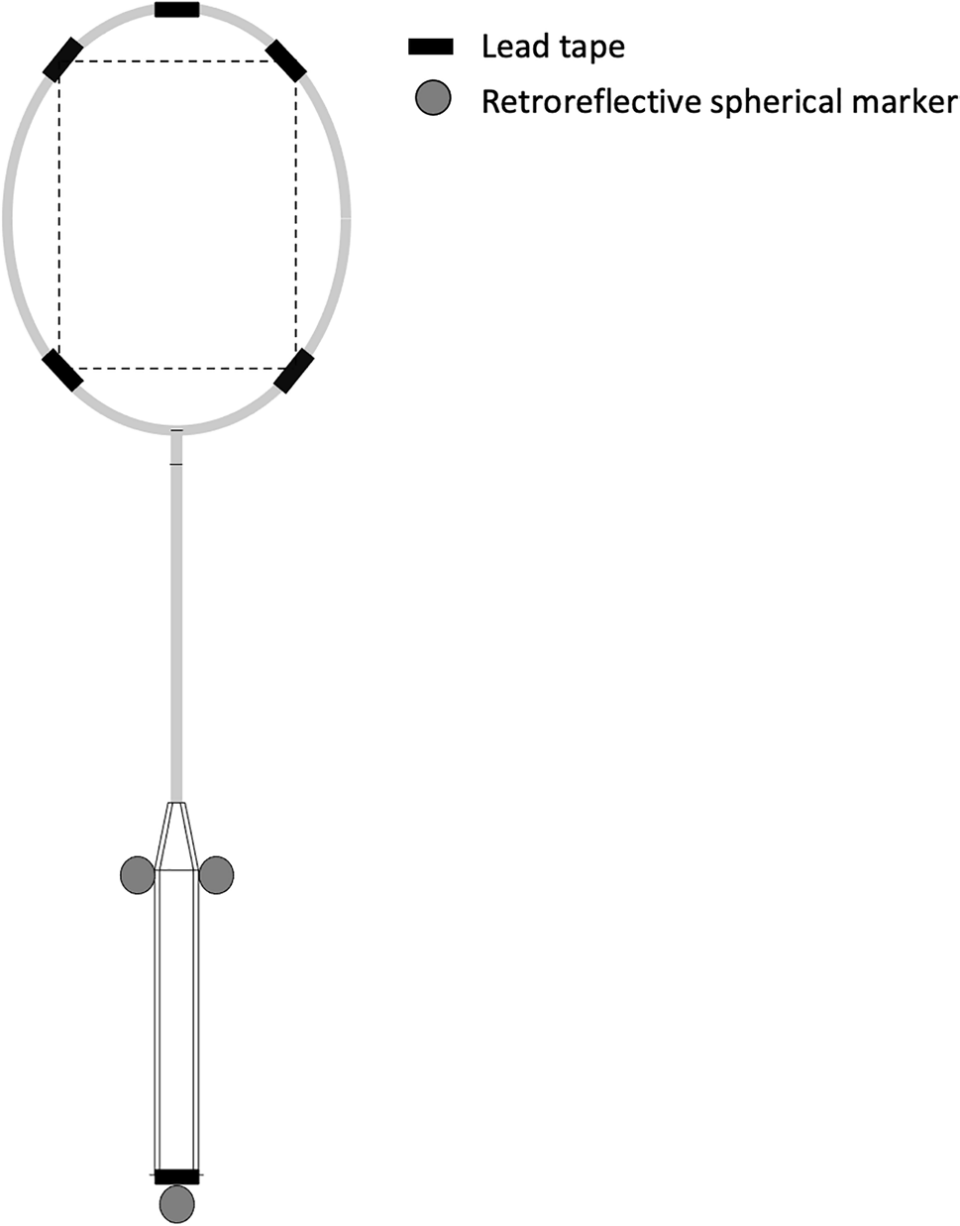
Application of lead tape to the rackets. Lead tape was covered in black tape so that it was not visually obvious to participants how the rackets were weighted. Depending on the desired ${I}_{s}$ value, lead tape was placed at either the racket tip or handle bottom.

Ten lightweight racket frames (mass = 70.4 ± 0.46 g, centre of mass = 31.16 ± 0.13 cm, ${I}_{s}$ = 73.5 ± 0.9 kg cm^2^) were customised to produce five rackets of equal mass but increasing ${I}_{s}$ by applying five pieces of lead tape (Stringers’ World, Essex, UK) per racket at specified points on the racket frame (Fig. 2). The amount of lead tape at each location was calculated based on the initial strung ${I}_{s}$ value of the racket, the desired overall mass and ${I}_{s}$, and keeping the amount of mass applied off-centre consistent across rackets to minimise the effects on ${I}_{P}$. All rackets had the same grip applied, and were strung by the same stringer, with the same string and tension (Yonex BG65ti, 28 lbs).

**Figure 3:**
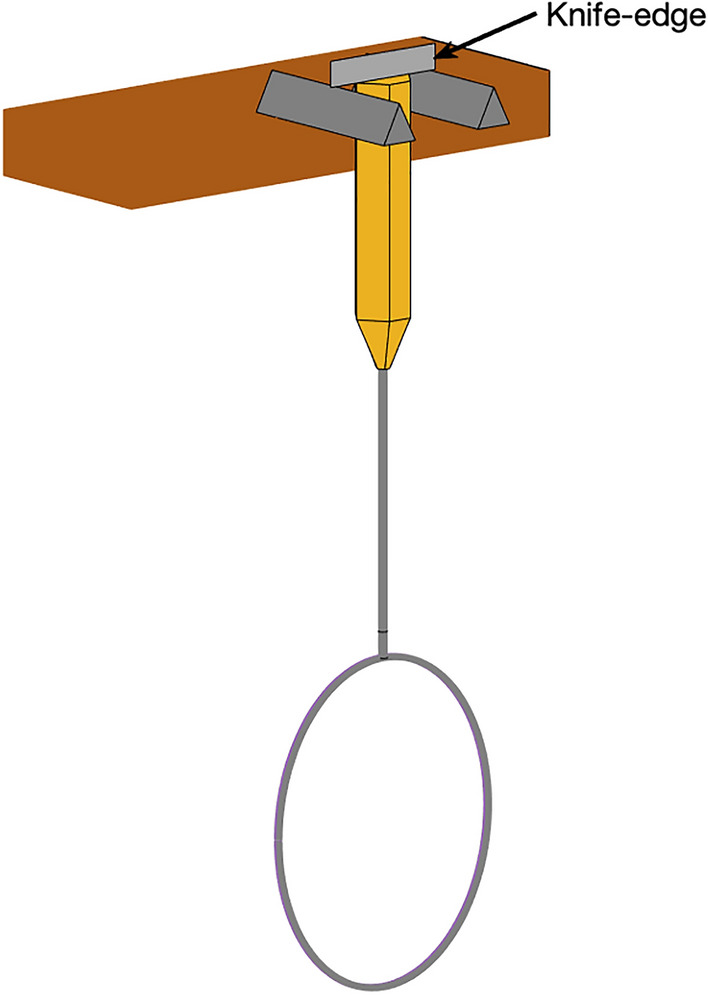
Schematic of the simple pendulum set-up.

Mass was calculated using a balance board with three scales, and centre of mass calculated by taking moments about the handle end. The MoI about the handle end (${I}_{h}$) was calculated using a simple pendulum method (Fig. 3; Eq. 1)^11–16^, and translated to the ${I}_{s}$ axis using the parallel axis theorem, Eq. (2)*.* The time of oscillation was measured using high-speed video (50 Hz), where the mean of fifty oscillations was taken as the time for a single oscillation ($T$), following previous recommendations^11^. This device was chosen due to it being lightweight and causing minimal friction. ${I}_{P}$ was determined using a moment of inertia measuring device (Inertia Dynamics, Connecticut, USA) where the racket was placed into a custom-build housing for the grip and was centralised using polystyrene inserts.

1 $$\begin{array}{c}{I}_{h}= \frac{{T}^{2}gmd}{4{\pi }^{2}}\end{array}$$

2 $$\begin{array}{c}{I}_{s}= {I}_{h}+mx\left(x-2d\right)\end{array}$$

where *g* is acceleration due to gravity (cm·s^−2^), $m$ is the mass of the racket (kg), $x$ is the distance from the handle end to the ${I}_{s}$ axis (cm) and $d$ is the distance from the handle end to the centre of mass (cm).

**Figure 4:**
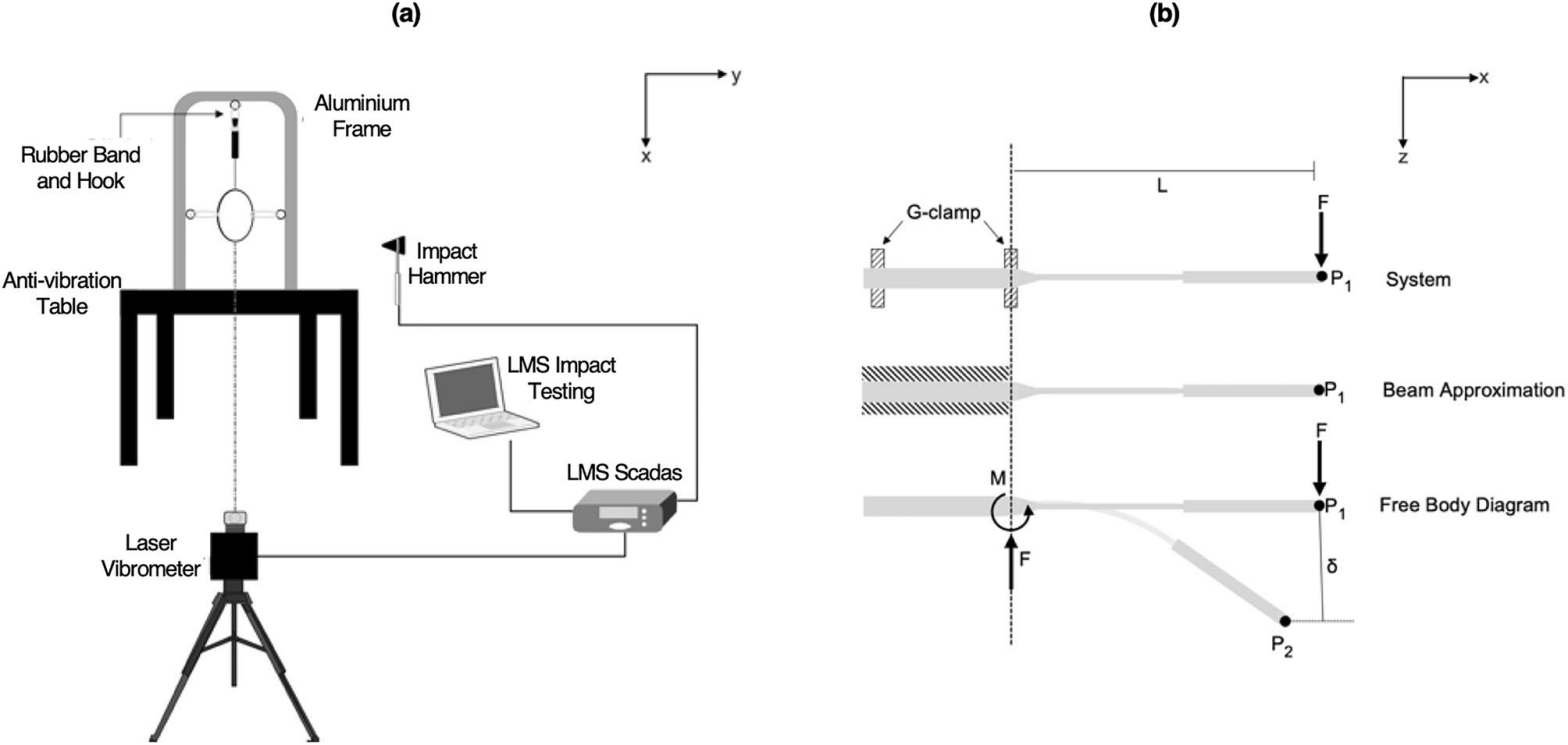
(**a**) Schematic of the experimental modal analysis set-up, (**b**) schematic of the beam approximation and load–deflection test.

For each racket, the fundamental frequency was determined using modal analysis, with the racket freely suspended and held in place by three rubber bands (assumed negligible mass and stiffness) at both sides of the racket head and the racket handle (Fig. 4a). The node location of the first bending mode (fundamental frequency) at the racket head end was determined by fitting a 2nd order polynomial to the modal data^17^. The repeatability of this protocol was measured by performing the experimental procedure five times for single racket in which the standard deviation of the fundamental frequency and node location were 0.1 Hz and 1.2 mm, respectively. Full details of this procedure are available in the supplementary materials.

A load–deflection test was used to quantify the *bending stiffness* of the racket perpendicular to the stringbed by attaching a mass of 1 kg to the tip of the racket and measuring the deflection at the racket tip (P_1_ to P_2_, z-direction). The racket was assumed encastre to model the frame as a beam (Fig. 4b). Assuming small deformations and linear-elastic material behaviour, the bending stiffness ($k$) of the racket frame can be calculated using Eq. (3):

3 $$\begin{array}{c}k=\frac{F}{\delta }\end{array}$$

where $F$ is the force applied at the racket tip, $\delta$ is the deflection (z-direction) following the application of force.

**Figure 5:**
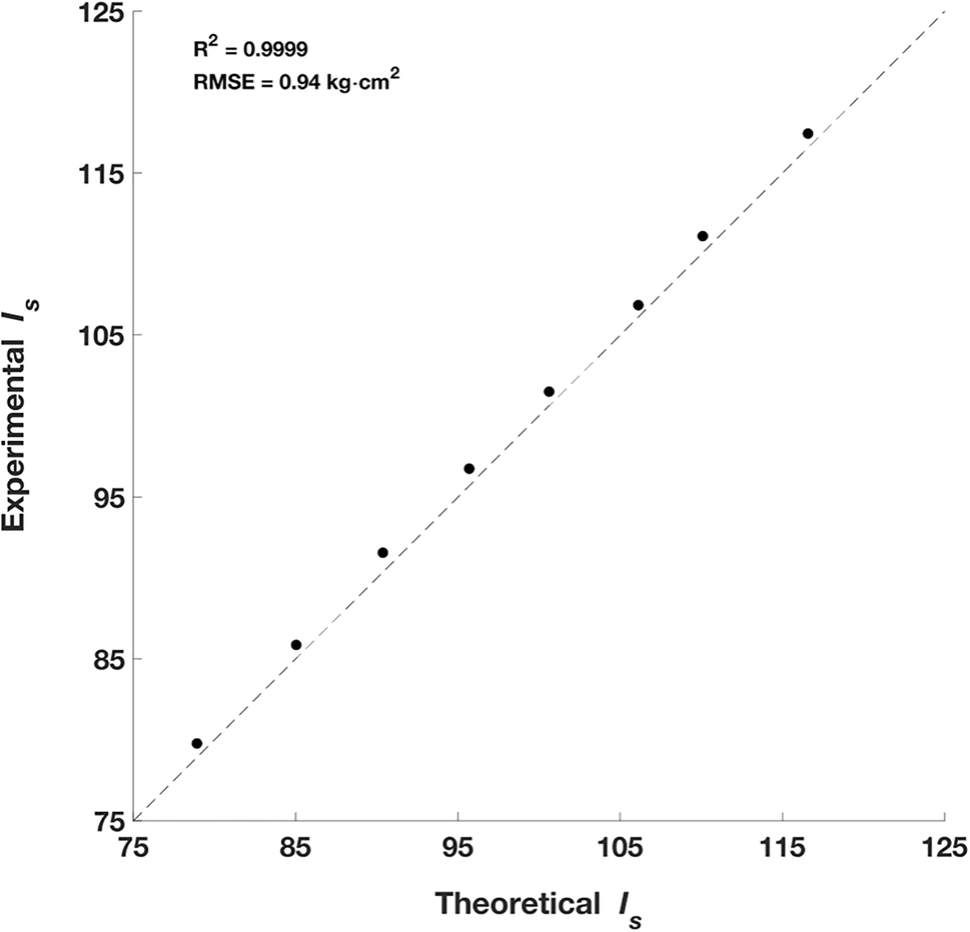
Theoretical vs. experimental ${I}_{s}$ of the eight calibration rods. The dashed line represents where the experimental and theoretical values are equal.

Accuracy of the ${I}_{s}$ calculation was determined by creating eight calibration rods that covered a range of 80–115 kg·cm^2^, inclusive of the typical range of badminton rackets^3^ (90–97 kg·cm^2^). When comparing theoretical and experimental ${I}_{s}$ values a systematic over-prediction of ${I}_{s}$ was found, however the agreement in the data was excellent (R^2^ = 0.9999, RMSE = 0.94 kg·cm^2^; Fig. 5). This offset was subtracted from all further calculations of ${I}_{s}$ in the study. The results validate the use of the pendulum method of accurately determining ${I}_{s}$ of implements within the region of 80–115 kg·cm^2^. The method is sensitive to small changes in centre of mass and time of oscillation, therefore precision in these values is necessary for accurate ${I}_{s}$ values. Accuracy of the racket mass properties were assessed using error propagation^18^, with mass, centre of mass and ${I}_{s}$ values accurate to ±  < 0.1%, 0.53% and 0.76%, respectively, which was deemed acceptable.

The study aimed to assess the effects of the ${I}_{s}$ in isolation, however due to the availability of rackets, it was decided to take a series of lightweight rackets and add mass to create incrementally different ${I}_{s}$ values, whilst keeping mass, bending stiffness and polar moment of inertia relatively constant and allowing the centre of mass to vary (Table 1). The modal response was affected with a reduction in the fundamental frequency (1st out-of-plane bending mode) and increase in distal node location (further from racket head centre) as ${I}_{s}$ increased (Table 1).

**Table 1:** Racket properties.

Racket	Mass properties				Stiffness properties			
	Mass (g)	COM (cm)	${I}_{s}$ (kg·cm^2^)	${I}_{P}$(kg·cm^2^)	Bending stiffness (N/m)	$\delta$(mm)	Fundamental frequency (Hz)	Node position (mm)
1	93.8	31.48	84.5	1.74	136.3	72.0	54.00	− 1.3
2	94.1	32.29	90.4	1.84	138.9	70.6	52.50	10.4
3	94.0	32.79	95.2	1.77	138.6	70.8	52.00	22.7
4	94.0	33.62	99.6	1.83	137.2	71.5	51.75	29.3
5	94.0	34.52	105.7	1.84	138.8	70.7	51.25	38.3

Node position refers to the first out of plane bending mode, on the frame, and is relative to the racket head geometric centre on the longitudinal axis, with a larger value indicating a node location closer to the racket tip. COM—centre of mass, $\delta$—the deflection during the load/deflection test.

## Stage 2: Effects of ${{\varvec{I}}}_{{\varvec{s}}}$ on badminton smash performance

### Data collection

20 (16 males; 4 females) experienced badminton players were recruited for this study and included players from regional to international standard (19.0 ± 3.7 years, 1.74 ± 0.07 m, 68.6 ± 8.6 kg). Paticipants performed a series of smashes using the five ${I}_{s}$ perturbed rackets along with their own racket and a commercially available racket. Testing procedures were explained to each participant, and informed written consent was obtained from participants or a parent/guardian, if under 18, in accordance with the guidelines of the Loughborough University Ethical Advisory Committee, from whom ethical approval was obtained.

Participants were instructed to complete a self-selected warm-up as if to prepare for competition. Racket order was determined using a Latin square design, such that every racket was represented in positions 1–5 equally and randomly assigned to participants to minimise possible order effects from learning and/or fatigue. Testing took place in a laboratory of sufficient height to allow players to produce their normal technique, as determined by an international badminton player. No a priori power analysis was performed, due to the minimum standard required for participants (regional), and therefore as many participants were recruited as possible.

**Figure 6:**
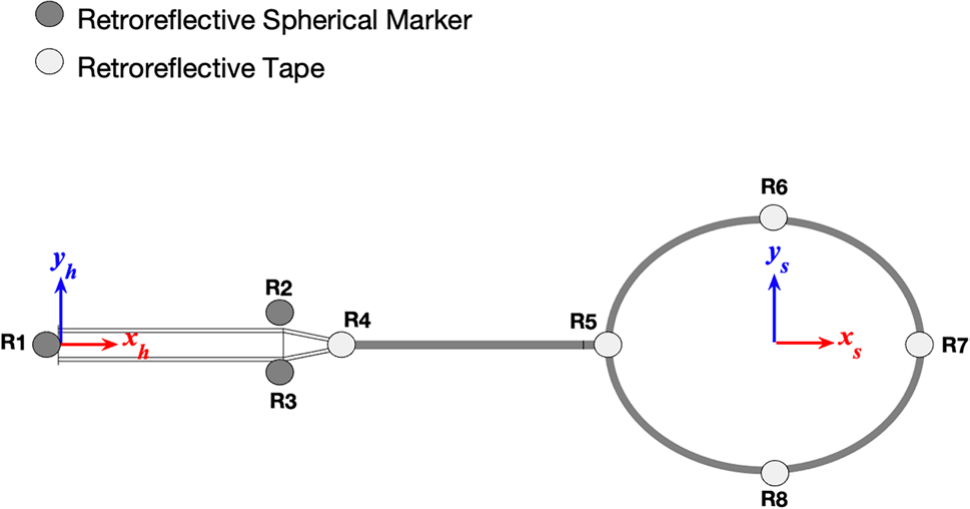
Schematic of the markers applied to racket, and the handle $\left({x}_{h},{ y}_{h}, { z}_{h}\right)$ and stringbed $\left({x}_{s},{ y}_{s}, {z}_{s}\right)$ coordinate systems, where both $z$ axes point towards the reader,

Retroreflective markers, both spherical and tape (Fig. 6), were applied to the racket, and were accounted for within the final reported ${I}_{s}$ value. Retroreflective tape was also applied to the base of the cork of the shuttlecock^1^. Three-dimensional position data of the racket and shuttlecock were captured using an 18-camera motion analysis system (Vicon OMG Plc, Oxford, UK) operating at 500 Hz.

To minimise learning effects further, participants performed a reduced set of trials with their own racket and a commercially available racket consisting of five maximal smashes each. Data were not captured for these trials. Participants then performed fifteen smashes (three sets of five) with each racket, with the shuttlecock launched every three seconds by a shuttlecock launcher (BKL, Badenko, France) to achieve consistency in the shuttlecock delivery, deemed representative of a lift stroke in competitive play by an international player. Participants were instructed to smash as fast as possible using their normal technique. Participants were permitted a one-minute rest between sets during each condition, as well as a five-minute rest between each condition. The number of smashes performed (85; including familiarisation) was representative of a normal training bout/competition for the level of the participant^19,20^.

### Data processing

All kinematic variables were calculated within a customised script in MATLAB v.2018b (MathWorks, Inc., Natick, USA). Shuttlecock speed, time of contact and thus pre- and post-impact phases were determined using a curve-fitting methodology^1^, where equations based on fundamental mechanical principles were applied to the global position data in the pre- and post-impact phases. The global coordinate system pointed to the participant’s right (x), towards the net (y) and vertical (z). The impact duration was assumed to be constant (1.4 ms), based on measurements using high-speed video (15,000 fps) when launching a shuttlecock at a freely suspended racket.

Racket head centre velocity was calculated using numerical differentiation of the racket head centre position data. Racket head speed was then calculated as the component of the racket head centre velocity acting in the instantaneous ${z}_{s}$-direction (Fig. 6) i.e., a racket moving solely in-plane would have a racket head speed of zero, with racket head speed at impact calculated by extrapolating the pre-impact data to a precise time of contact. To differentiate between the component of racket head speed caused by the grip motion (i.e., the theoretically rigid-body motion component) and the component due to racket deformation with respect to the grip, a virtual rigid racket head centre was created by projecting a point 0.348 m, determined during static trials, in the ${x}_{h}$-direction (Fig. 6) from R4. The deformation contribution towards racket head speed was then calculated as the racket head speed minus the virtual rigid racket head centre speed. A justification for using the stringbed coordinate system is given in the Limitations section.

The impact location was determined by expressing the shuttlecock position within the stringbed plane and relative to the racket head geometric centre^1^. To calculate an impact location at the precise time of impact, cubic polynomials were fit to pre-impact racket head marker (R5, R6, R7, R8) position data, and the shuttlecock position defined using the previously described curve-fitting methodology.

To represent a participant’s performance with each racket, with respect to racket head speed and shuttlecock speed, the mean of the fastest three trials for each participant was calculated. To establish a typical impact location with each racket, the first five trials were removed to eliminate effects of familiarisation^5^, and the mean of the remaining ten trials used.

### Statistical analysis

Statistical analyses were performed within SPSS v.27.0 (IBM Corp., Armonk, NY, USA). The effects of ${I}_{s}$ on racket head speed, deformation behaviour (minimum deformation and deformation velocity at impact), shuttlecock speed and impact location were assessed using a one-way, repeated measures analysis of variance (ANOVA), with a significance threshold of *p* < 0.05. Effect sizes were determined using eta-squared (${{\eta}}^{2}$) and interpreted as: *large* ≥ 0.14; 0.14 > *medium* ≥ 0.06; 0.06 > *small* ≥ 0.01; *trivial* < 0.01^21^. Data were assessed for normality (Shapiro–Wilk test) and sphericity (Mauchly’s test) with a Greenhouse–Geisser correction being applied when the assumption of sphericity was violated. For statistically significant main effects, Bonferroni post-hoc pairwise t-tests were performed to determine where the differences between conditions existed.

## Results

**Figure 7:**
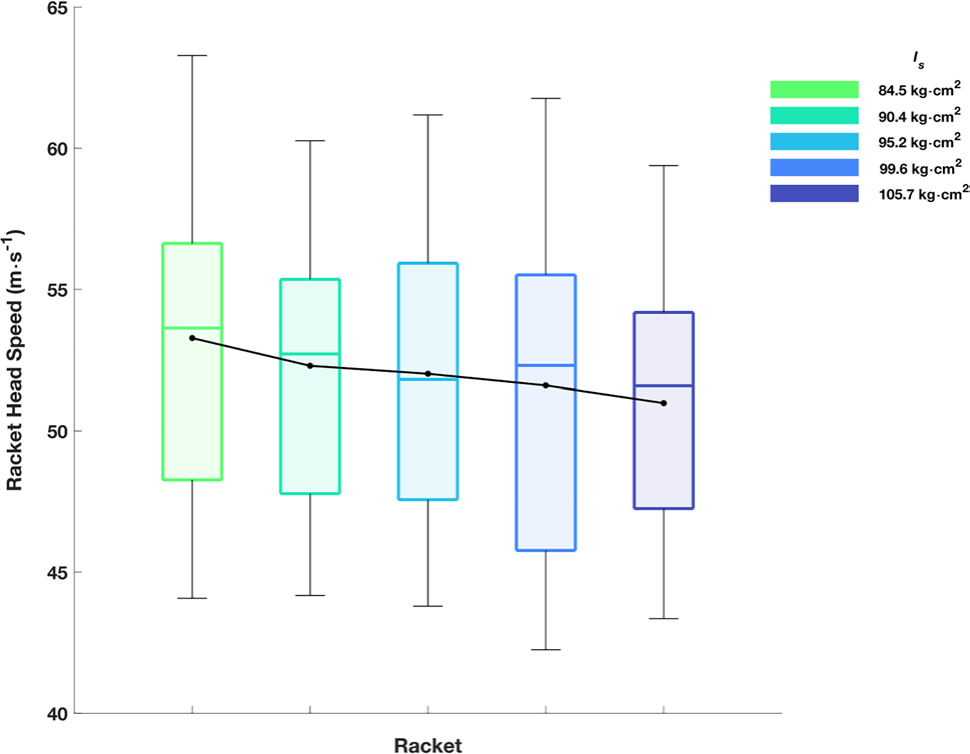
Racket head speed for each racket. The box plots represent the interquartile range (shaded area), median (solid coloured line), minimum/maximum (whiskers). The solid black line and circles represent the mean.

**Figure 8:**
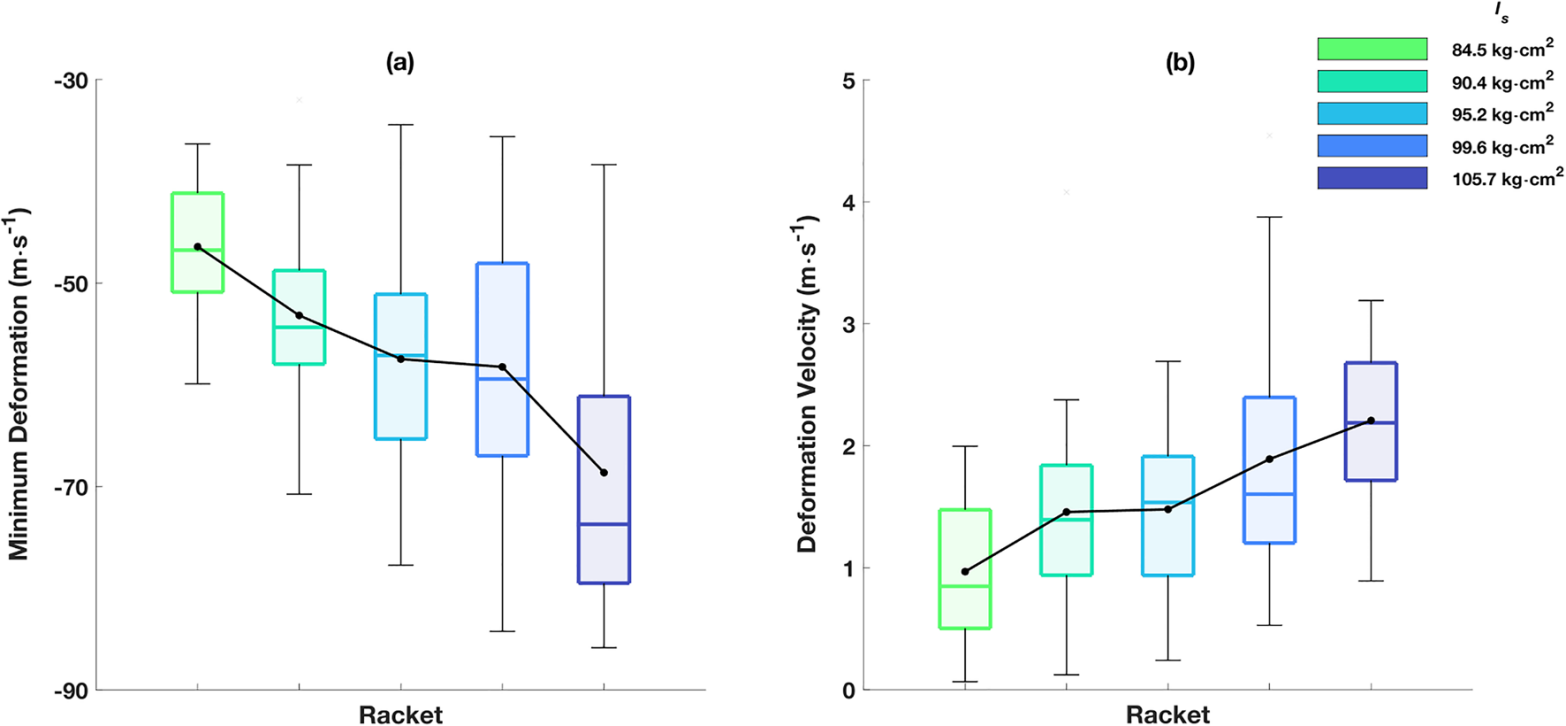
(**a**) Maximum deformation for each racket, (**b**) deformation velocity at impact for each racket. The box plots represent the interquartile range (shaded area), median (solid coloured line), minimum/maximum (whiskers). The solid black line and circles represent the mean.

Racket head speeds achieved were 52.0 ± 5.3 (42.2–63.3) m·s^−1^ for all 1500 trials (300 per racket). A *large* significant main effect was found (F_(4, 76)_ = 10.41,* p* < 0.001, $\eta^{2}$ = 0.354; Fig. 7) highlighting an inverse relationship between ${I}_{s}$ and racket head speed. Post-hoc tests revealed significant differences for five out of ten pairwise comparisons. Focussing on the rigid component of racket head speed increased the effect size (F_(4,76)_ = 18.60, *p* < 0.001; $\eta^{2}$ = 0.493). Furthermore, rackets of greater ${I}_{s}$ produced more deformation (F_(4,76)_ = 24.46, *p* < 0.001; $\eta^{2}$ = 0.565; Fig. 8a) and greater deformation velocities (F_(4,76)_ = 20.11, *p* < 0.001; $\eta^{2}$ = 0.514; Fig. 8b), i.e., greater moments required to accelerate higher moments of inertia generate larger deformation magnitudes and results in observed higher recovery (deformation) velocities despite lower natural frequencies.

**Figure 9:**
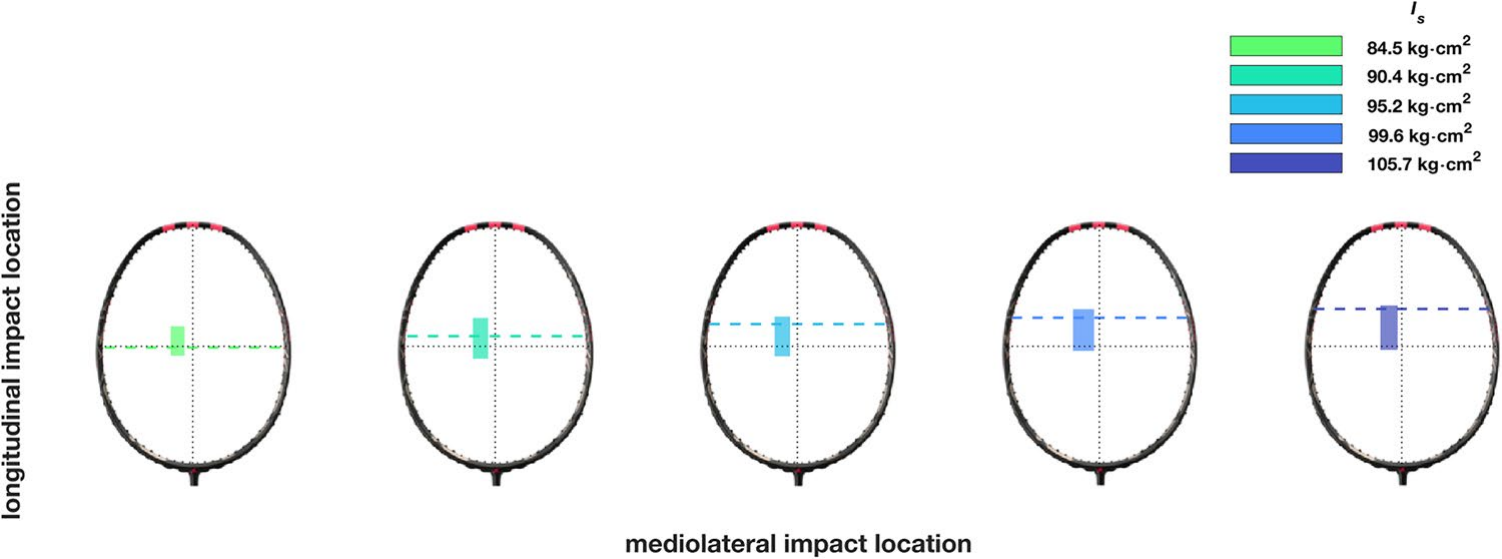
Impact locations for each racket. The shaded area represents the mean ± SD in both the mediolateral and longitudinal directions, respectively. The dashed line represents the distal node locations of the first bending mode.

A *large* significant main effect was found (F_(4, 76)_ = 3.33,* p* = 0.014, $\eta^{2}$ = 0.149; Fig. 9) highlighting a positive relationship between ${I}_{s}$ and longitudinal impact location. Post-hoc testing revealed no significant differences between conditions. No significant main effect was found between ${I}_{s}$ and mediolateral impact location (F_(4, 76)_ = 0.70,* p* = 0.594, $\eta^{2}$ = 0.036).

**Figure 10:**
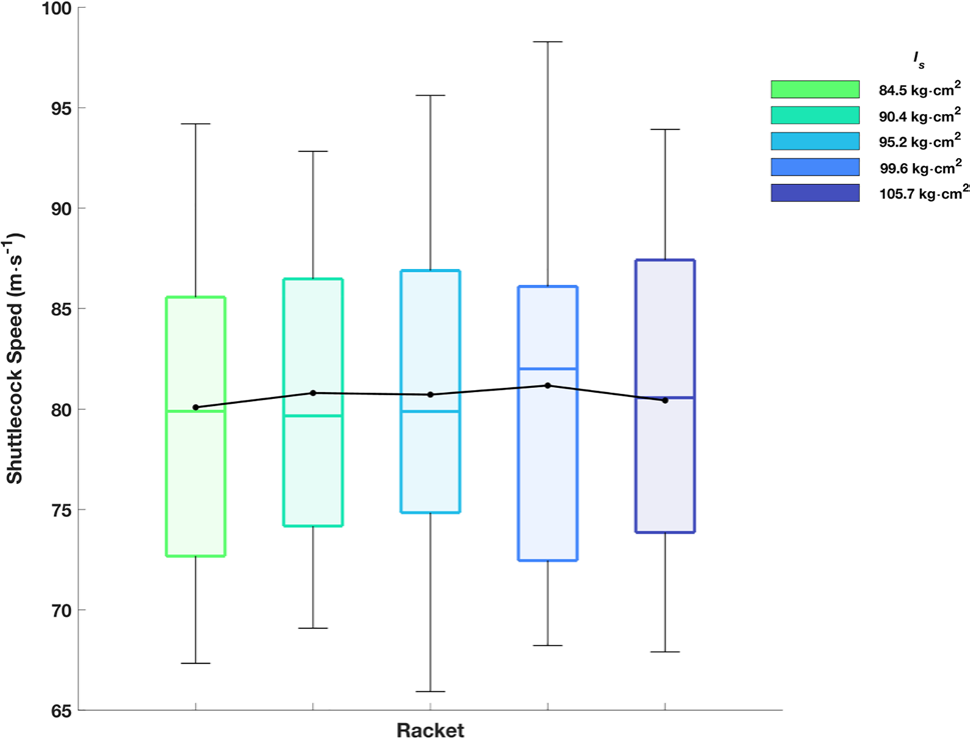
Shuttlecock speed for each racket. The box plots represent the interquartile range (shaded area), median (solid coloured line), minimum/maximum (whiskers). The solid black line and circles represent the mean.

Participants averaged shuttlecock speeds of 80.5 ± 8.2 (65.9–98.3) m·s^−1^. No significant main effect was found with respect to ${I}_{s}$ (F_(4, 76)_ = 0.56,* p* = 0.696, $\eta^{2}$ = 0.028; Fig. 10).

## Discussion

The results suggest that whilst an increase in ${I}_{s}$ caused lower racket head speeds and a distal increase in longitudinal impact location, there was no effect on the post-impact shuttlecock speed. Shuttlecock speeds were similar to those reported by previous researchers^1,2,22^ for players of a similar standard.

There was a significant inverse relationship between ${I}_{s}$ and racket head speed at the racket head centre, with a *large* effect size (*p* < 0.001; $\eta^{2}$ = 354). It is possible that the effect of ${I}_{s}$ on racket head speed was compensated for by an increased deformation component with increasing ${I}_{s}$, due to addition of greater mass nearer the tip of the racket causing a reduction in natural frequency. Focusing on the rigid component of racket head speed produced a larger effect size (*p* < 0.001; $\eta^{2}$ = 0.493).

The fastest racket head speeds (racket head centre) calculated in this study were greater than previous literature^3,23^: 64 m·s^−1^ vs. ~ 53 m·s^−1^ and ~ 45 m·s^−1^. This was most likely due to the ability of the participants and not methodological differences, as sampling frequencies were very similar to the present study: 480^3^ and 500^23^ Hz, respectively. Kwan et al*.*^23^ recruited three participants of varying skill level: elite (Division 1, National Taiwan College Cup), sub-elite (Division 2, National Taiwan College Cup) and recreational. Kwan^3^ recruited five participants (three advanced and two novice). The present study included players that could be considered advanced (regional) up to players regularly competing on the Badminton Europe circuit (elite).

It is also possible that the instrumentation added to the racket caused lower racket head speeds by increasing mass and ${I}_{s}$: Seven retroreflective markers increasing mass by 13.2 g (from 89.2 g to 102.4 g), decreasing balance point by 12.1 mm, and increasing ${I}_{s}$ by 6.48 kg·cm^2^ (from 89.8 to 96.2 kg·cm^2^)^23^. Kwan^3^ did not state whether their reported ${I}_{s}$ value included the addition of eight retroreflective markers thereby possibly underestimating mass and ${I}_{s}$, which could explain the slower racket head speeds. The present study added three spherical retroreflective markers near the ${I}_{s}$ axis whilst tape markers added negligible mass. The addition of this marker set increased ${I}_{s}$ by only 0.51 kg·cm^2^ and was accounted for in the final mass and ${I}_{s}$ value. The present study concludes that whilst tape markers attached to the racket head may be tracked less accurately, the smaller impact on mass and ${I}_{s}$ make it a favourable method to reproduce realistic racket properties used by players.

**Figure 11:**
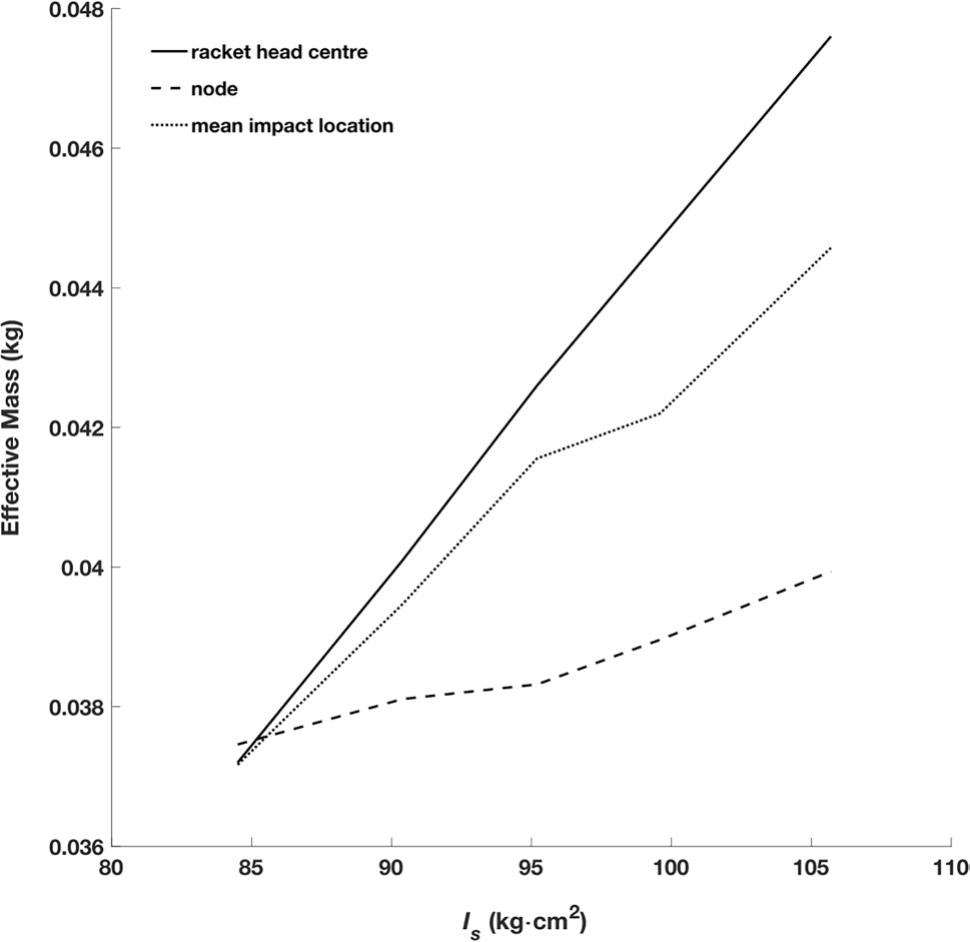
Effective mass of impacts at the racket head centre, node location and mean impact location. All impacts assume an impact on the longitudinal axis (mediolateral impact location = 0).

The effect seen with racket head speed was not present when focusing on post-impact shuttlecock speed, where no difference was detected. Additionally, whilst racket head speed decreased as ${I}_{s}$ increased, on average players’ longitudinal impact location was greater (more distal) with higher ${I}_{s}$ rackets. This has previously been attributed to the loss of effective mass with lower ${I}_{s}$ rackets (Fig. 11) and possibly impact efficiency being worth the gain in racket head speed at the impact location, due to an increased arc length (racket handle to impact location)^3^.

Previous research has found this variation in impact location in both badminton^3^ and tennis^5^. A *large* effect size ($\eta^{2}$ = 0.514) was found for increasing longitudinal impact location with increasing ${I}_{s}$ during the tennis serve (noting a small difference in the ${I}_{s}$ definition for tennis rackets)^5^. The greater effect could be because the tennis serve is a relatively closed skill where the player has control of the incoming ball trajectory as well as it being a much slower speed than that of an incoming shuttlecock, where the opposing player dictates the speed and trajectory of the shuttlecock, causing larger variation in impact location. Additionally, as the badminton racket is typically moving faster than a tennis racket, with racket head speeds achieved of over 60 m·s^−1^ for elite players, it is unsurprising that there is larger variation in impact location, with standard deviations of 9 and 20 mm (Fig. 9) in the medio-lateral and longitudinal directions, respectively, compared to 11 and 10 mm in tennis^5^.

Two possible explanations for this phenomenon exist, related to the node location and the inertial properties of the racket. Firstly, players may be tuning their timing and technique such that the impact location occurs closer to the node location of the first bending mode, which moves closer to the tip as more mass is placed at the tip^9^. Two main advantages exist as a result of an impact location closer to the node location: firstly, the mode associated with the node point (first bending mode) is excited less, and therefore vibrations of this frequency are felt less by the hand and humans are haptically sensitive to vibrations at frequencies between 50 and 200 Hz^24^, where typical fundamental frequencies of badminton rackets are in the region of 50–55 Hz. Research in tennis has suggested that, consciously or subconsciously, players aim for the node location^7,8^. Secondly, less energy is lost (through vibration of the racket) when the racket makes contact with the shuttlecock at the node point, and the majority of the vibration amplitude derives from the lower order modes^25^.

A second explanation for these differences in impact location as a function of ${I}_{s}$ could be due to inertial properties of the racket, where it could be hypothesised that rackets with more mass at the tip and greater ${I}_{s}$ results in the tip travelling lower and as a result the shuttlecock impacts higher on the stringbed which still provides good ‘feel’ and is not consciously or subconsciously compensated for. Whilst a significant increase in longitudinal impact location was found with increasing ${I}_{s}$, no post-hoc tests were significant, and as such a conclusive statement cannot be made and further research is required with different research designs where rackets of similar inertial properties, but differing node locations could be used.

Of more relevance may be the individual, as opposed to group, responses to ${I}_{s}$ perturbations. Whilst the general trend was for racket head speed at the racket head centre to decrease by ~ 0.7 m·s^−1^ for every ~ 5 kg·cm^2^ increase in ${I}_{s}$, individually the racket head speeds could be up to 5 m·s^−1^ different between rackets and sometimes producing greater racket head speed values with higher ${I}_{s}$ rackets. This could be due to numerous reasons including increased deformation velocity for higher ${I}_{s}$ rackets, more familiarity with higher ${I}_{s}$ rackets and technique. It should be noted that the inverse linear relationship was not present for some of the players, where the greatest racket head speeds were often produced with Rackets 2 and 3, perhaps due to unfamiliarity with very low ${I}_{s}$ rackets, where Rackets 2 and 3 were more representative of a commercial range^4^. The relationship between ${I}_{s}$ and racket head speed may therefore be an inverse linear or inverse quadratic relationship within the range used in this study, dependent upon the individual.

Previous studies have used a power law, $V=C/{{I}_{s}}^{n}$, to describe the relationship between ${I}_{s}$ and implement speed, where $C$ is a player constant and $n$ is the power of the relationships^26^. The *n* values averaged 0.22 ± 0.13, which were lower than the 0.32 reported by Kwan^3^. Similar *n* values have been reported using softball bats (0.25)^27^, golf clubs (0.19)^28^ and tennis (0.31)^9^. It is not possible to establish the existence of a universal power law dependent on ${I}_{s}$, as a large amount of variation across participants was evident (− 0.05–0.45), as well as no clear trends where previous research has suggested that more skilled players display lower *n* values^3^. For badminton rackets, a linear relationship between racket head speed and ${I}_{s}$ may not be appropriate given that the moment of inertia of a badminton racket and hand about the handle end would be close to 150 kg·cm^2^ and racket head speed will not become infinite as ${I}_{s}$ approaches zero^29^. Many players’ racket head speeds are lower with the lowest ${I}_{s}$ racket perhaps due to unfamiliarity and perhaps lack of confidence at this range.

## Limitations

Appropriate methodological steps were taken to ensure that tracking of the racket and shuttlecock were accurate, using filtering and curve-fitting. The use of retroreflective tape was prioritised over spherical markers due to adding negligible mass. Ideally, the racket-shuttle contact period would have been measured for every trial; however, this was not possible in a whole-body capture volume and an assumed 1.4 ms contact period from high-speed video was used. The effect of this was negligible, where a 0.1 ms decrease in contact time caused, on average, a 1 mm decrease, a 0.5 mm decrease and a 0.13 m·s^−1^ increase in longitudinal impact location, mediolateral impact location (more medial) and post-impact shuttlecock speed, respectively. This represents 0.4%, 0.3% and 0.2% of the range.

Using the vector normal to the stringbed coordinate (${z}_{s}$) system to represent racket head speed was justified given that no differences in deformation orientation at impact were found between the rackets (F_(4, 76)_ = 0.54,* p* = 0.700), typically between 5–10 mm behind neutral at impact i.e., the racket had not fully recovered. The use of markers on the handle (R2 and R3) were in very close proximity and as such were unable to represent the orientation of the grip (rigid-body motion) in comparison to the head markers (R6 and R8) where an adequate distance was present. Instrumentation added to the grip, such as rods with markers affixed to the ends, to allow the true orientation of the grip to be quantified would have hindered the natural swing of the participants and affected the inertial properties of the rackets.

The node location was based upon values of a freely suspended racket which is a better representation of in-play conditions^24^, however, in reality, the hand-gripped condition will alter the node location, typically moving the node towards the tip and downwards at the handle^18^. Additionally, the nodal line is not straight, and the node is more longitudinally distal on the frame compared to the stringbed, making the nodal line curved^24,30^. The accuracy of the modal analysis was not assessed, only the repeatability, however fundamental frequencies were similar to previously reported values of 56–62 Hz^31,32^, noting that other research has reported the clamped fundamental frequency^23,33,34^.

Further work could evaluate the repeatability of the protocol used in this study. Testing whether the effects occur with different racket orders would allow an understanding of the consistency of the relationships found. Previous research in golf tested the effects of moment of inertia on clubhead and ball velocity where participants took part in multiple data collections each with a different club order, providing more confidence in the results^6^

Finally, it should be noted that the smash stroke is one of many strokes within badminton, and that racket selection to optimise performance (shuttlecock speed) of one stroke may be detrimental to other strokes. For example, if for a given player a racket of high ${I}_{s}$ is determined as optimal, this may have negative consequences for defensive strokes or attacking net strokes in which greater manoeuvrability and racket head acceleration is required.

## Conclusions

In conclusion, an inverse relationship was found between ${I}_{s}$ and racket head speed, however this did not lead to a reduction in shuttlecock speed, likely due to an increase in effective mass for a given impact location and more distal longitudinal impact locations as ${I}_{s}$ increased. The more distal impact locations, as ${I}_{s}$ increased, coincided with an increase in the location of the distal node of the first bending mode, which may have been due to players subconsciously seeking a desirable ‘feel’ during impact i.e., minimise vibration within a range where humans are haptically sensitive. Future studies should confirm the coincidence between node location and impact location by removing inertial effects i.e., rackets with equivalent mass properties but varied modal properties, and additionally consider adaptations to moment of inertia over a prolonged period of time as opposed to the acute effects. Other performance factors such as shot accuracy or fatigue could also be evaluated, as ${I}_{s}$ is perturbed. Additionally, player perception data should be measured, where it would be expected that shot quality/feeling would be negatively correlated with distance between the impact location and node location.

General findings do not negate the possibility of there being an individual optimum that this protocol could be used to determine. Whilst the overall group response to changes in ${I}_{s}$ caused no change in shuttlecock speed, for certain individuals, selection of a racket of a particular ${I}_{s}$ value could lead to improved performance.

### Supplementary Information

Supplementary Information.

## Data Availability

The datasets generated during and/or analysed during the current study are not publicly available due to commercial restrictions but are available from the corresponding author on reasonable request.
