# Supplementary material for: Effects of racket moment of inertia on racket head speed, impact location and shuttlecock speed during the badminton smash
Source: Sci Rep. 2023 Aug 28;13:14060. doi: 10.1038/s41598-023-37108-x (PMC10462755; doi:10.1038/s41598-023-37108-x)
Supplement: Supplementary file 1 — Supplementary Information. [file 41598_2023_37108_MOESM1_ESM.docx]

**Effects of racket moment of inertia on racket head speed, impact location and shuttlecock speed during the badminton smash**

H. Towler^1*^, S.R. Mitchell^2^, M.A. King^1^

# Supplementary Materials

*Moment of Inertia*

The moment of inertia about the handle end ($I_{h}$) was determined using a simple pendulum method (Figure 1) similar to Spurr *et al.*^11^*.* A small lightweight device was attached to the handle end, comprising a polystyrene block exposing two ends of a knife edge that was free to oscillate about the edges of two triangular supports. The time of oscillation was measured using high-speed video (50 Hz), where the mean of fifty oscillations was taken as the time for a single oscillation ($T$), following previous recommendations^11^. $I_{P}$ was determined using a moment of inertia measuring device (Inertia Dynamics, Connecticut, USA) where the racket was placed into a custom-build housing for the grip and was centralised using polystyrene inserts.


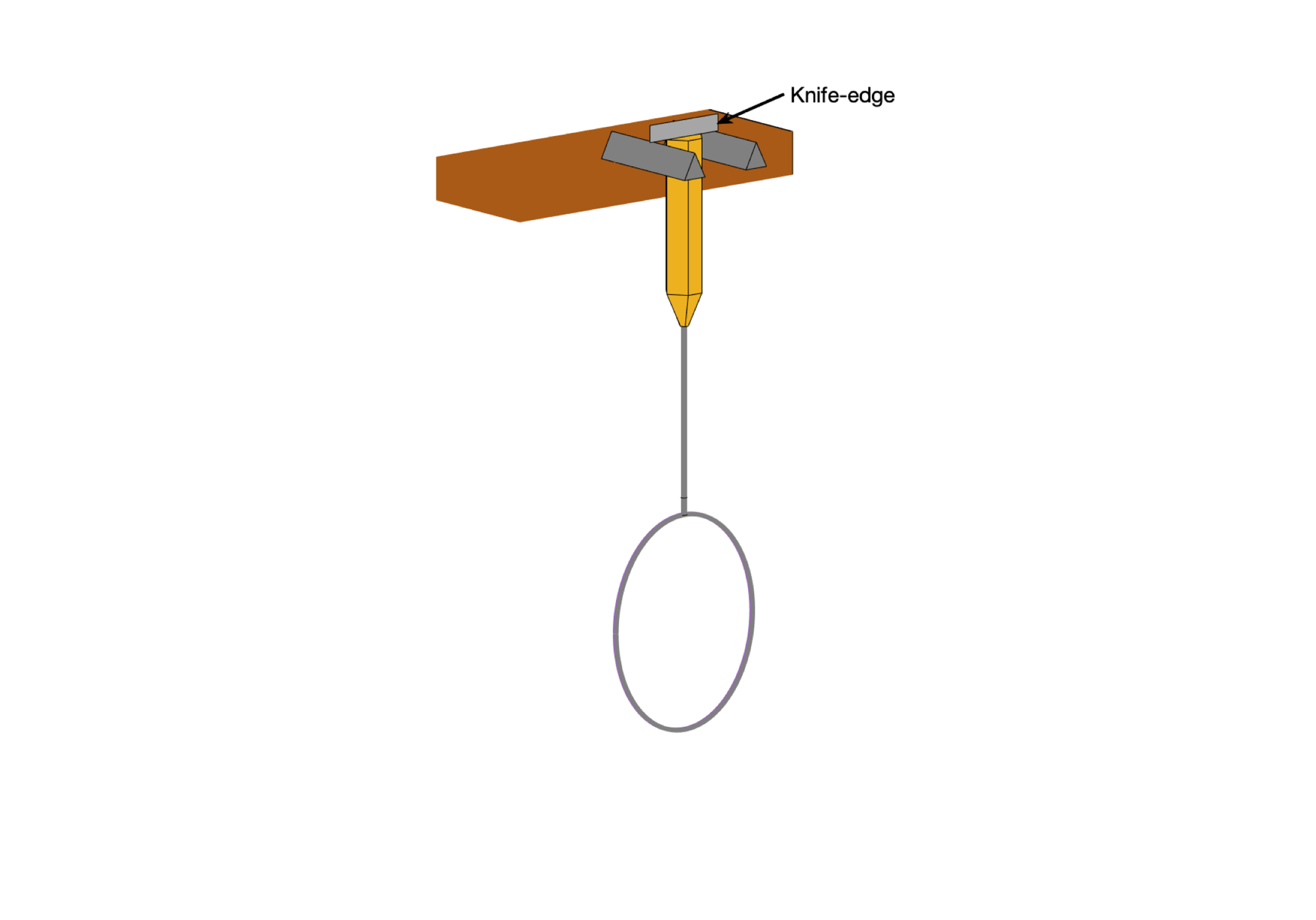


Figure 1. Schematic of the simple pendulum set-up.

$I_{h}$ was calculated using Equation (1) and converted to the $I_{s}$ axis using the parallel axis theorem, Equation (2). This device was chosen due to it being lightweight and causing minimal friction.

$\begin{aligned} I_{h}= \frac{T^{2}gmd}{4\pi^{2}} \#(1) \end{aligned}$

$$\begin{aligned} I_{s}= I_{h}+mx\left( x-2d \right)\#\left( 2 \right) \end{aligned}$$

where *g* is acceleration due to gravity (cm·s^-2^), $m$ is the mass of the racket (kg), $x$ is the distance from the handle end to the $I_{s}$ axis and $d$ is the distance from the handle end to the centre of mass (cm).

To determine the accuracy for calculating $I_{s}$, eight calibration rods were fashioned by cutting aluminium rods (Ø = 7.95 mm) to specified lengths to produce eight different theoretical $I_{s}$ values, that covered a range typical of badminton rackets, 90-97 kg·cm^2^, previously reported^12^. MoI about the end of the rod ($I_{r}$) was calculated and translated to the $I_{s}$ axis, Equation (2), with uniform density assumed such that the centre of mass was positioned at the geometric centre. Experimental (simple pendulum method) and theoretical values were then compared.

A systematic over-prediction of $I_{s}$ was found, however the agreement in the data was excellent (R^2^ = 0.9999, RMSE = 0.94 kg·cm^2^; Figure 2). This offset was subtracted from all further calculations of $I_{s}$ in the study. The results validate the use of the pendulum method of accurately determining $I_{s}$ of implements within the region of 80-115 kg·cm^2^. The method is sensitive to small changes in centre of mass and time of oscillation, therefore precision in these values is necessary for accurate $I_{s}$ values. Accuracy of the racket mass properties were assessed using error propagation^13^, with mass, centre of mass and $I_{s}$ values accurate to ± <0.1%, 0.53% and 0.76%, respectively, which was deemed acceptable.


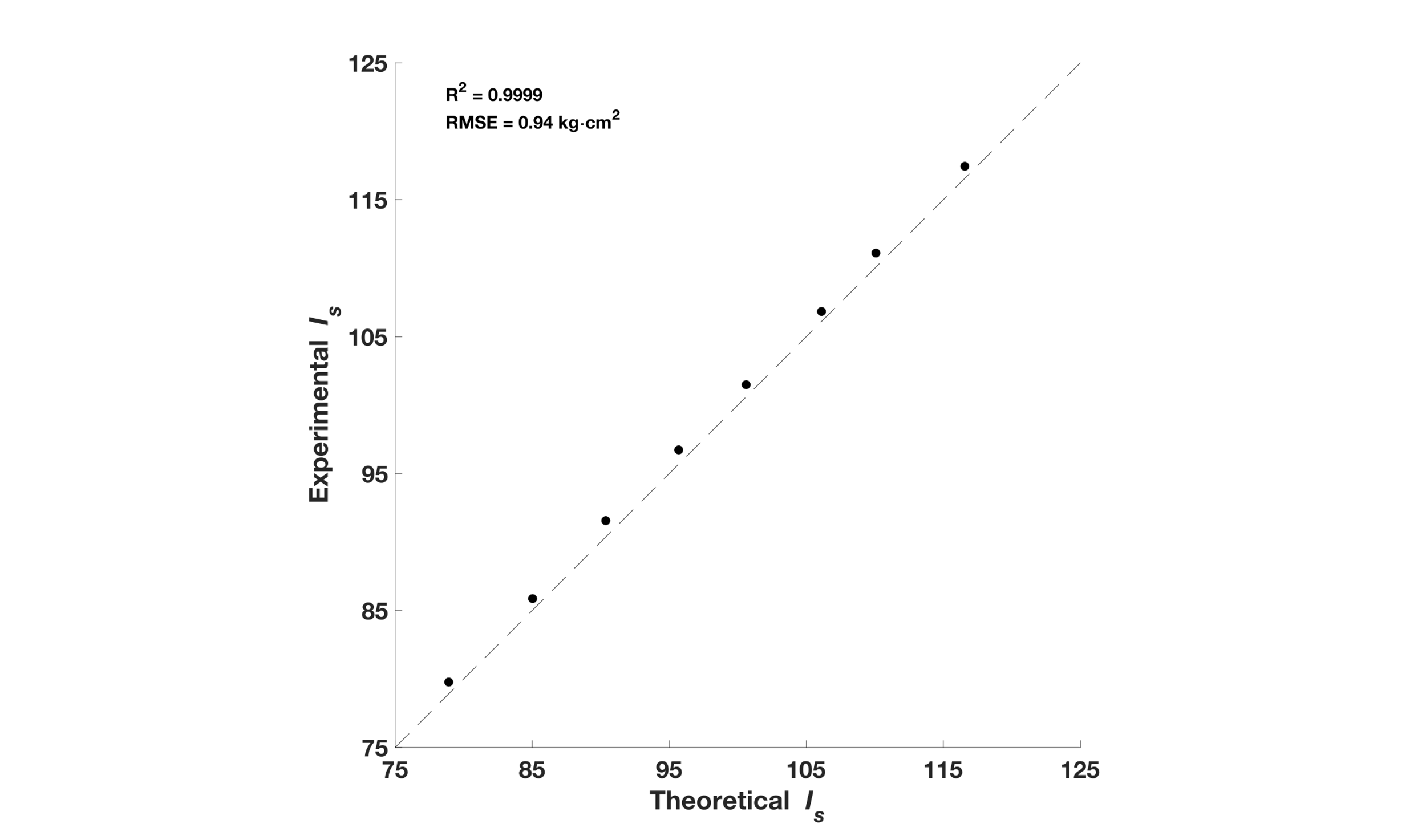


Figure 2. Theoretical vs. experimental $I_{s}$ of the eight calibration rods. The dashed line represents where the experimental and theoretical values are equal.

*Modal Analysis*

For each racket, the fundamental frequency was determined using modal analysis, with the racket freely suspended and held in place by three rubber bands (assumed negligible mass and stiffness) at both sides of the racket head and the racket handle (Figure 3a). The input was captured with an impact hammer (Brüel & Kjær, Type 8206-001) fitted with a steel tip, and a single-point laser vibrometer (Polytec PDV 100) to measure the response. Data were acquired using an LMS Scadas frontend and LMS Impact Testing software. Linear averaged frequency response functions (FRF) were calculated from 10 individual measurements, each acquired over a duration of 4 s with a 0.18 s pre-trigger at a sampling frequency of 2.048 kHz with a useful bandwidth of 1.024 kHz, allowing a resolution of 0.25 Hz. The node location of the first bending mode (fundamental frequency) at the racket head end was determined by fitting a 2^nd^ order polynomial to the modal data (Glynn *et al.,* 2011). The repeatability of this protocol was measured by performing the experimental procedure five times for single racket in which the standard deviation of the fundamental frequency and node location were 0.1 Hz and 1.2 mm, respectively.

Figure 3. (a) Schematic of the experimental modal analysis set-up, (b) schematic of the beam approximation and load-deflection test

*Bending Stiffness*

A load-deflection test was used to quantify the *bending stiffness* of the racket perpendicular to the stringbed by attaching a mass 1 kg to the tip of the racket and measuring the deflection at the racket tip (P_1_ to P_2_, z-direction) using a motion capture system (Vicon; 500 Hz). The racket was clamped to a workbench at the base of the top cap and at the bottom of the handle using two G-clamps (Figure 3b) and was assumed encastre to model the frame as a beam. Assuming small deformations and linear-elastic material behaviour, the bending stiffness ($k$) of the racket frame can be calculated using Equation (3):

$$\begin{aligned} k=\frac{F}{\delta} \#(3) \end{aligned}$$

where $F$ is the force applied at the racket tip, $\delta$ is the deflection (z direction) following the application of force.
